# Gastropleural Fistula as a Rare Complication of Gastric Sleeve Surgery: A Case Report and Comprehensive Literature Review

**DOI:** 10.1155/2018/2416915

**Published:** 2018-12-20

**Authors:** Fahad Alghanim, Ali Alkhaibary, Abdulmohsen Alzakari, Abdullah AlRumaih

**Affiliations:** ^1^College of Medicine, King Saud bin Abdulaziz University for Health Sciences, Riyadh, Saudi Arabia; ^2^Department of Thoracic Surgery, King Abdulaziz Medical City, Riyadh, Saudi Arabia; ^3^Department of Surgery, King Abdulaziz Medical City, Riyadh, Saudi Arabia

## Abstract

Gastropleural fistula (GPF) is a rare, life-threatening complication of gastric sleeve surgery. GPF is an uncommon differential diagnosis to consider in a patient presenting with a picture of pneumonia. As such, GPF should be suspected in a patient with a history of nonresolving pneumonia who recently underwent gastric sleeve surgery. To the best of our knowledge, only eight cases of gastropleural fistulas after bariatric surgery have been reported in the literature. Herein, we report a case of gastropleural fistula after gastric sleeve surgery and review the pertinent literature. A gastropleural fistula is an exceedingly rare and life-threatening complication postbariatric surgery. Nonsurgical conservative management (total parenteral nutrition, percutaneous drainage, and antibiotics with endoscopic stenting) can be considered.

## 1. Introduction

Laparoscopic sleeve gastrectomy (LSG) is a restrictive, nonreversible bariatric procedure for managing obesity. It involves removing 85% of the stomach and stabling back the remaining portions together. The long-term consequences of LSG, however, on the overall health and mortality remain uncertain [1].

Although LSG is a relatively safe surgical option for weight loss, some complications have been reported in the literature. Such complications include, but are not limited to, leakage, clots, infections, strictures, and hemorrhage, with gastric sleeve dilatations and staple-line leaks being the most common [2].

Gastric leaking, by definition, is the leak of luminal contents from a surgical join between two hollow viscera [3]. In the vast majority of patients, the duration of gastric leak development is usually less than 14 days after bariatric surgery [4]. Patients may be asymptomatic or present with signs and symptoms of septic shock. Patients usually present with a sudden abdominal pain, accompanied by fever and tachycardia [3].

Considering the rarity of gastropleural fistulas, this case report outlines the clinical presentation, radiological findings, and outcome of a 24-year-old male who was diagnosed with a gastropleural fistula that is communicating with a perisplenic collection after gastric sleeve surgery.

## 2. Case Description

A 24-year-old male presented with a history of nonresolving pneumonias. Nine months prior to presentation, the patient was severely obese (body mass index = 41 kg/m^2^) for which he underwent gastric sleeve surgery. Thereafter, his weight decreased from 108 kg to 54 kg.

Four months prior to presentation, the patient was having a whitish, productive cough and was diagnosed with pneumonia which was treated with oral antibiotics. The patient then presented to the emergency department with severe shortness of breath for four days, left-sided noncardiac chest pain, and weight loss.

Physical examination revealed a decreased air entry in the left lung, scattered rhonchi, generalized body pain, and fever. The first impression was that of pneumonia.

The patient was initially admitted under pulmonology with a picture of left lung pneumonia with pulmonary effusion. The patient had a pigtail insertion in the left side, for which pleural fluid was sent for cultures. The patient was assessed and examined by thoracic surgery, and the decision was made for a left chest decortication.

**Figure 1:**
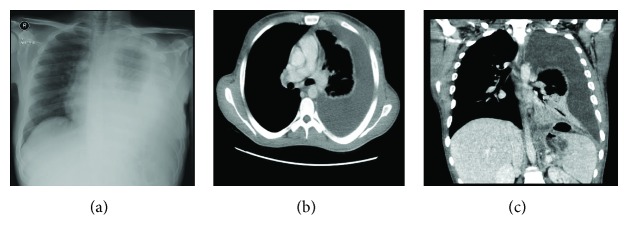
(a) Posterio-anterior chest radiograph showing a large left pleural effusion with atelectasis of the left lung. (b) Axial chest CT showing a large left pleural effusion with complete atelectasis of the left lower lobe. (c) Sagittal abdominal and pelvis CT showing a large leak with perisplenic collection. The abdominal collection is connected to the pleural collection through a fistula.

Preop imaging showed a large gastric leak with perisplenic collection (Figure 1). The collection was communicating with the upper pole of the spleen. There was a questionable pleural fistula at the collection. Therefore, the surgery was aborted, and an upper GI endoscopy was requested.

The chest CT revealed a partly loculated, large left pleural effusion with complete atelectasis of the left lower lobe and partial atelectasis of the left upper lobe. Few alveolar and tree-in-bud opacities were noted in the right upper lobe and apical segment of the right lower lobe, representing pulmonary infections (Figure 1(b)).

An 8-French pigtail drainage catheter was inserted into the left pleural cavity, and the infected empyema was aspirated. Then, the abdominal and pelvis CT showed a large leak, with perisplenic collection measuring 2.5 × 5.5 × 5.7 cm. This collection was communicating with the other collection seen at the upper pole of the spleen that measured 4 × 4.2 cm. The pleural collection measured 1.8 × 2 × 4.2 cm. The abdominal collection was connected to the pleural collection through a fistula (Figure 1(c)).

**Figure 2:**
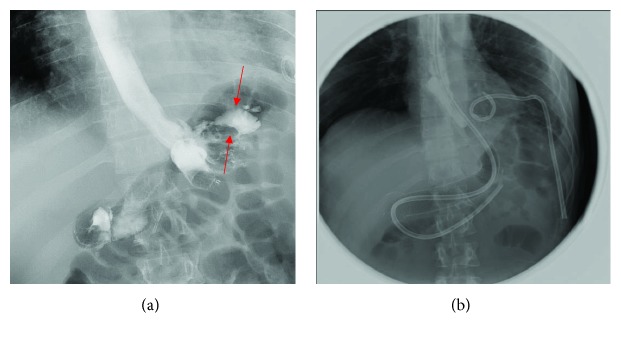
(a) Upper GI series showing frank extravasation of the oral contrast at the site of the proximal suture (red arrows). (b) Upper GI series showing no gastric leak upon comparison with the previous upper GI study.

The upper GI endoscopy showed a large fistula coming out of the stomach and the upper boarder of the gastric cardia. The upper GI series showed a frank extravasation of the oral contrast at the site of the proximal suture (Figure 2(a)).

A covered esophageal stent and nasojejunal tube were inserted to control the gastric leak. Through a transoral approach, a wire and a 5F catheter were used to canalize the remaining part of the stomach. Contrast was given which showed a leak at the left lateral aspect. Then, the catheter over the wire was passed into the bowel reaching to the jejunal loop, followed by an exchange to a stiffer wire. Subsequently, an esophageal stent, measuring 24 mm × 23 cm, was successfully placed.

Eight days later, another upper GI series was performed which showed no gastric leak upon comparison with the previous upper GI study (Figure 2(b)). The guidewire and catheter passed down the esophageal stent, which was completely retrieved using crocodile forceps under fluoroscopy. An Esophogram demonstrated an initial hold-up of contrast at the distal esophagus. Contrast eventually passed through the stomach into the small bowel.

Prior to discharge, repeated imaging showed an improvement of the left-sided pleural effusion and splenic collection. Therefore, the patient was discharged in a stable condition with antibiotics and pain killers. Follow-up in the clinic after 3, 6, and 15 months of the diagnosis confirmed that the patient was medically free with no active issues.

## 3. Discussion

Gastropleural fistula is a rare complication by which the stomach lumen is pathologically communicating with the pleural space. The first case of gastropleural fistula was described by Markowttz and Herter in 1960 [5]. To date, multiple etiologies of gastropleural fistulas have been described in the literature; one of which is bariatric surgery [6, 7]. Sleeve gastrectomy has been particularly identified to be one of the causes accounting for the development of gastropleural fistulas [8].

The presentation of gastropleural fistulas is usually insidious with patients being clinically stable. Nonetheless, the clinical presentation is variable, as some patients might present in an unstable condition shortly after a sleeve gastrectomy. Symptoms might include shortness of breath, chest pain, cough, recurrent respiratory infections, fever, or abdominal pain [6].

In accordance with what has been previously reported in the literature (Table 1), the patient in the present case presented 9 months after gastric sleeve surgery with shortness of breath, productive cough, chest pain, and recurrent pulmonary infections. A gastropleural fistula was suspected, and, therefore, the appropriate diagnostic investigations were performed to confirm the diagnosis.

Of note, there is a huge debate in the literature on how patients with gastropleural fistulas should be managed, due to the absence of guidelines and scarcity of case reports. Most reports show that a laparoscopic approach might yield better outcomes [9]. However, conservative management with antibiotics, parenteral nutrition, percutaneous drainage of collections, and endoscopic therapies showed varying results [10–12]. One study presented two cases that were managed by a robotic approach concluding that the utility of the robotic platform in such complex surgical cases is safe and feasible [7].

It is evident that cases of gastric fistulas have been increasingly reported especially in the context of sleeve gastrectomies to address severe degrees of obesity. Therefore, the treatment strategies for such cases have been continuously evolving [13]. In the present case, the patient was managed conservatively with antibiotics, abscess drainage, and placement of an esophageal stent. The stent was not removed until the 8th week, resulting in a stenosis that was managed with balloon dilatation.

## 4. Conclusion

Gastropleural fistulas are exceedingly rare and life-threatening complications postbariatric surgery. The signs and symptoms pointing to gastropleural fistulas can be misleading and nonspecific. As such, surgeons should keep a high index of suspicion to identify patients with recurrent respiratory tract infections after bariatric surgery. Nonsurgical conservative management (total parenteral nutrition, percutaneous drainage, and antibiotics with endoscopic stenting) might be considered.

## Figures and Tables

**Table 1:** Summary of the reported cases of gastropleural fistula in the literature.

No.	1st author	Age	♀/♂	Presentation	Duration to fistula after bariatric surgery	Management	Outcome
1	Jiramethee [14]	61	F	Worsening dyspnea & fever	2 months	Not available	Successful repair of the dehiscence
2	Garcia-Quintero [7]	24	F	Recurrent pneumonia, fever, and cough	11 months	Laparoscopic robotic-assisted esophagogastrectomy with Roux-en-Y reconstruction	Stenosis of the esophagojejunal anastomosis after 4 months
3	Garcia-Quintero [7]	57	M	Cough, hemoptysis, & flank pain	13 years	Partial gastrectomy, resection of the gastropleural fistula, hiatal hernia repair, & decortication	Discharged after 4 days, with an uneventful follow-up
4	Ghanem [15]	43	M	Abdominal pain	6 months	Enteric stent insertion to cover the fistula opening	Resolution of the fistula after two months, symptom-free after 5 months
5	Al-Shurafa [6]	37	F	Dry cough, chest pain, & dyspnea	2 years	Excision of the fistula & repair of the diaphragmatic defect	Discharged home in an excellent condition
6	Nguyen [8]	41	M	Presented for revisional surgery	9 months	Side-to-side esophagojejunostomy & jejunojejunostomy	Well after 5 months
7	Ladd [16]	25	F	Recurrent pneumonia	2 months	Over-the-scope clip at the gastric opening & endoscopic approach	Discharged 2 days postoperatively
8	Andrawes [17]	54	F	Septic shock	11 years	Endoscopic suturing & esophageal stent placement	Resolution of the fistula after 6 months
9	Present case	24	M	Productive cough, dyspnea, & recurrent pneumonia	9 months	Total parenteral nutrition, percutaneous drainage, & antibiotics with endoscopic stenting	Discharged in a stable condition with antibiotics and pain killers

## References

[1] Franco J. V. A., Ruiz P. A., Palermo M., Gagner M. (2011). A review of studies comparing three laparoscopic procedures in bariatric surgery: sleeve gastrectomy, Roux-en-Y gastric bypass and adjustable gastric banding. *Obesity Surgery*.

[2] Lalor P. F., Tucker O. N., Szomstein S., Rosenthal R. J. (2008). Complications after laparoscopic sleeve gastrectomy. *Surgery for Obesity and Related Diseases*.

[3] Rached A. A., Basile M., El Masri H. (2014). Gastric leaks post sleeve gastrectomy: review of its prevention and management. *World Journal of Gastroenterology*.

[4] Csendes A., Braghetto I., León P., Burgos A. M. (2010). Management of leaks after laparoscopic sleeve gastrectomy in patients with obesity. *Journal of Gastrointestinal Surgery*.

[5] Markowttz A. M., Herter F. P. (1960). Gastro-pleural fistula as a complication of esophageal hiatal hernia. *Annals of Surgery*.

[6] Al-Shurafa H., Alghamdi S., Albenmousa A., Alolayan H., Al-Shurafa Z. (2017). Gastropleural fistula after single anastomosis gastric bypass. A case report and review of the literature. *International Journal of Surgery Case Reports*.

[7] Garcia-Quintero P., Hernandez-Murcia C., Romero R., Derosimo J., Gonzalez A. (2015). Gastropleural fistula after bariatric surgery: a report of two cases. *Journal of Robotic Surgery*.

[8] Nguyen D., Dip F., Hendricks L. S., Lo Menzo E., Szomstein S., Rosenthal R. (2016). The surgical management of complex fistulas after sleeve gastrectomy. *Obesity Surgery*.

[9] Mehran A., Ukleja A., Szomstein S., Rosenthal R. (2005). Laparoscopic partial gastrectomy for the treatment of gastropleural fistula. *Journal of the Society of Laparoendoscopic Surgeons*.

[10] Marr B., Needleman B., Mikami D. (2012). Endoscopic stenting for treatment of leaks following sleeve gastrectomy. *World Journal of Laparoscopic Surgery*.

[11] Kunieda T., Sakata N., Yamakita N. (2012). Gastropleural fistula. *Internal Medicine*.

[12] Alazmi W., Al-Sabah S., Ali D. A. M., Almazeedi S. (2014). Treating sleeve gastrectomy leak with endoscopic stenting: the Kuwaiti experience and review of recent literature. *Surgical Endoscopy*.

[13] Trelles N., Gagner M., Palermo M., Pomp A., Dakin G., Parikh M. (2010). Gastrocolic fistula after re-sleeve gastrectomy: outcomes after esophageal stent implantation. *Surgery for Obesity and Related Diseases*.

[14] Jiramethee N., Mira-Avendano I., Phelan J. (2017). An unusual case of gastro-pleural fistula masquerading as pneumonia following bariatric surgery. *American Journal of Respiratory and Critical Care Medicine*.

[15] Ghanem O. M., Abu Dayyeh B. K., Kellogg T. A. (2017). Management of gastropleural fistula after revisional bariatric surgery: a hybrid laparoendoscopic approach. *Obesity Surgery*.

[16] Ladd A. M., Al-Bayati I., Shah P., Haber G. (2015). Endoscopic closure of a gastropleural fistula. *Endoscopy*.

[17] Andrawes S., El Douaihy Y. (2017). Using the endoscopic overstitching device and fully covered esophageal stents for closure of a gastropleural fistula and repair of a deformed gastric sleeve. *VideoGIE*.

